# Chemical Recycling of Plastics by Microwave‐Assisted High‐Temperature Pyrolysis

**DOI:** 10.1002/gch2.201900074

**Published:** 2020-02-14

**Authors:** Haibin Jiang, Wenlu Liu, Xiaohong Zhang, Jinliang Qiao

**Affiliations:** ^1^ SINOPEC Beijing Research Institute of Chemical Industry 14, Beisanhuan Donglu, Chaoyang District Beijing 100013 China; ^2^ College of Materials Science and Engineering Beijing University of Chemical Technology 15, Beisanhuan Donglu, Chaoyang District Beijing 100029 China

**Keywords:** carbon, chemical recycling, graphene, microwave

## Abstract

Use of plastics faces much criticism because of its shocking and increasing impact on the environment. But banning plastic will not help the environment. Only appropriate recycling of plastic waste can give satisfying solution. A lab‐scale chemical recycling method, in which a mixture of plastic wastes and plant oil is continuously cracked into ethylene, propylene, and other useful chemicals by using microwave‐assisted high‐temperature pyrolysis, is developed. The method has delivered interesting leads that provide the basis for setting up a new process. Based on the encouraging results, a “drop‐in” method for a renewable and circular polymer industry is also proposed. If it is commercially realized, plastic waste and plant oils will be the feedstock for the polymer industry and this industry will become renewable and circular.

## Graphical abstract

With the new microwave‐assisted pyrolysis process, plant oils and plastic wastes are cracked into monomers, which can be polymerized into plastics again with the help of existing petrochemical technologies. This new process can achieve two things by one stroke: renewable and circular, a promising solution for both biorefinery and circular plastic industry.

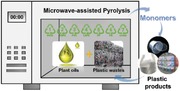

Plastics are indispensable materials in modern economy. It was reported that 8.3 billion tons virgin plastics were produced from 1950s to 2017 and 6.3 billion tons have become plastic wastes. Unfortunately, only 9% plastic wastes have been recycled and 12% incinerated.[qv: 1] The environmental consequences of plastic solid waste are visible in ever‐increasing levels both on land and in the oceans.[qv: 2] In addition, it has been proved that human bodies are becoming polluted with plastic.[qv: 3] Nowadays, there is no retreat in the face of plastic pollution as it is one of the greatest environmental challenges. Campaigners have called for reductions or bans on plastics in many countries. However, an outright ban on plastics could cause even more harm to the environment and lead to triple the amount of greenhouse gas emissions because manufacturing of alternate materials such as glass and metal will double energy consumption.[qv: 4] The consensus is that only suitable recycling technology for plastic waste can give satisfying solution to plastic pollution.

Therefore, tremendous efforts have been devoted to plastic recycling for decades. Plastic recycling is currently divided into physical recycling and chemical recycling. Physical recycling, also called mechanical recycling, has been widely adopted for large‐scale treatment of sorted plastic solid waste. The treated and recycled plastic, usually of a single type, is often blended with virgin plastic of the same type to produce a material with suitable properties for manufacturing.[qv: 2] Nowadays, poly(ethylene terephthalate) (PET) and polyethylene (PE), which represent 9% and 37%, respectively, of the annual plastic consumption, have been successfully recycled by this process. Yet, chances are the product quality will be impaired by some degradable species existing in the recycled plastic.[qv: 5] Further, in addition to high cost, water pollution, and downgrade in application, temperature‐sensitive plastics, composites and plastics that do not flow at elevated temperatures such as thermosets, cannot be recycled by this process. By comparison, pyrolysis developed to recover the chemical products from plastics in the presence of a catalyst, referred to as a chemical recycling process, is considered to move beyond physical recycling.[qv: 2,6] But harsh pretreatment steps are still often required to protect the catalyst.[qv: 7] Therefore, relatively pure waste polymer feedstock is needed, which requires costly and time‐intensive sorting and pretreating of municipal solid waste.[qv: 2] Even though various chemical recycling without catalysts, such as thermal processes, do not require pure waste polymers, the resulting products are often oils due to the relatively low temperature. Obviously, the current recycling technologies are far from solving the problem of plastic pollution at a reasonable cost; therefore, we need scientific breakthrough to recycle plastics economically. Herein, we report a new lab‐scale chemical recycling method based on a new finding, in which a very high temperature is produced to avoid the use of catalyst in pyrolysis. This approach delivered interesting outcomes and provided the basis for developing a new process.

The new chemical recycling method cracks plastics into olefins such as ethylene and propylene at high temperature without using any catalyst. Recently, it has been reported[qv: 8] that graphene could produce large arcs during microwave irradiation, which typically lasted for 50–100 ms per arcs. The ultrafast heating process upon arcs could generate very high temperature up to 1000 °C or even several thousands of Celsius in only tens of milliseconds, which is extremely suitable for cracking hydrocarbon into olefins at high temperatures with short residence time. Therefore, graphene foam (GF) (see Figure S1, Supporting Information) was prepared according to the previous work.[qv: 9] A total of 0.5 g linear low‐density PE (LLDPE) film was then put on 0.2 g GF. The GF with LLDPE film sealed in nitrogen was heated in a household microwave oven at 700 W for 40 s. Thereafter, the gas was taken by a 100 mL syringe for gas chromatography (GC) analysis. Surprisingly, all solid PE films were cracked into gases that consisted of ethylene, propylene, and other useful chemicals, such as methane and hydrogen. The result was similar to that of traditional chemical recycling, although catalyst was not used. Other most consumed plastics were also examined by using the same method, including scraps of PE terephthalate (PET) bottle, high‐density PE bottle cap, polypropylene (PP) pellets, and polystyrene (PS) foam. The results were similar to that of LLDPE film; all plastic scraps vanished under intensive arcs in 40 s, as shown in Figure S2, Supporting Information. Based on the above new finding, it is possible to develop a technology for plastic recycling with commercial value if two major challenges can be overcome: to find a reasonable material to replace the expensive graphene and to develop a continuous process.

In order to know which materials can replace graphene, it is important to know how microwave‐excited arcs are formed. According to literature,[qv: 10] they are essentially the discharges when potential differences are large enough to break down the electrical resistance of the medium. Specifically speaking, the microwave‐induced current on the surface resonates with the microwave frequency; the current and the microwave frequency are not at the same speed. The time required for the complete polarization is very short, typically of the order of 10^−18^ s. Moreover, a significant potential differences may form; because the conductive electrons are extremely mobile, while in a microwave cavity, the time required for the applied electric field to reverse completely is far longer than this, ≈2 × 10^−10^ s at 2.45 GHz. Based on the above mechanism, it is possible to substitute graphene by other carbon materials. Carbon nanotube foam (CNTF) (see Figure S3, Supporting Information), as an alternative carbon material, was used to replace graphene foam for cracking plastic wastes and similar results and phenomenon to that of GF were obtained. Considering CNTF is also expensive and not renewable, water‐soluble starch, which is cheap and renewable, was used as carbon precursor, and a starch‐based carbon cuboid was used to replace GF for PE film cracking. Unfortunately, the PE film was just slightly melted instead of being cracked into gas after 700 W microwave irradiation for 40 s. The result was similar to that of a previous publication,[qv: 11] in which particulate carbon (particles <300 µm) was used to crack plastic wastes under microwave irradiation. With a maximum power output as high as 5 kW, the carbon particles were only heated to 700 °C at best, and the pyrolysis products were mainly liquid. It is obvious that a carbon material with porous structure is indispensable to obtain microwave‐induced high temperature; therefore, a plate composed of aluminum oxide fiber (AF) was used as a robust porous skeleton. The pore widths of AF were about tens of microns (see Figure S4, Supporting Information), which were similar to those of GF and CNTF. According to the mechanism of arc forming mentioned above, proper pore structure of carbon material is very important for heating to high temperature under microwave irradiation. The discharges will not occur as long as the pores are too wide to break down. The energy released by the discharge will not be as high as required for such high temperature when the widths are too small due to the relatively low potential differences causing the break down. Carbon‐coated AF (CAF) (see Figure S4, Supporting Information) was obtained by carbonizing the dried starch‐coated AF. All plastic scraps vanished under intensive arcs in 40 s as well when CAF was used instead of GF or CNTF. Thus, a renewable starch‐based carbon material with controlled porous structure can also be used as a heating material for this new plastic recycling method.

A continuous process was designed for pyrolysis, as it is well known that a continuous process is much more efficient than a batch process for large‐scale application. As shown in **Figure** **1**, the mixture of plant oil and plastic powder as feedstock was continuously pumped into microwave cavity through a quartz tube and pyrolyzed to gas by microwave‐excited CAF. After condensation, the pyrolysis gas was collected for GC analysis. A thermocouple was used to detect the temperature of the microwave‐excited CAF. With 900 W microwave and 30 g CAF in the cavity, the temperature reached 800 °C in just 3 min and reached more than 1000 °C in 7 min, as shown in Figure S5, Supporting Information. In fact, the heating rate of CAF should be much higher than the curve shown, because the heat capacity of the metal thermocouple will delay the temperature of the test.

**Figure 1:**
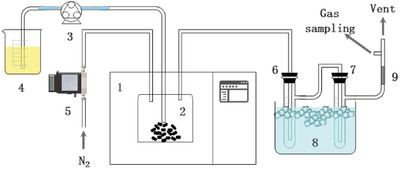
Schematic diagram of the experimental setup for the continuous pyrolysis process. (1) Microwave oven; (2) quartz reactor; (3) peristaltic pump; (4) feedstock; (5) gas flowmeter; (6, 7) cold traps; (8) ice‐water bath; and (9) cotton wool filter.

Plant oils, as important renewable resources, usually cannot be cracked into gas by using traditional steam‐cracking process because the steam temperature is not high enough. However, this new microwave‐excited CAF can reach more than 1000 °C, and all plant oils that were tried were cracked into gas. For example, palm oil was continuously pyrolyzed by using the new process as shown in Figure 1 at 900 W microwave. The products consisted of 71.4 wt% gas, 22.0 wt% solid, and 6.6 wt% liquid. The gas contained 30.3 wt% ethylene, 4.5 wt% propylene, and other useful chemicals (3.3 wt% hydrogen, 15.5 wt% carbon monoxide, and 24.1 wt% methane). The detailed gas composition is listed in **Table** **1**. The solid and liquid products are biocarbon and condensed polycyclic aromatic hydrocarbons, respectively. When the mixture of palm oil and plastic are used as feedstock, better results are obtained.

**Table 1:** Products of different feedstocks with the continuous pyrolysis

Products/wt%	Palm oil	POPE	POPP
Solid phase	22.0	14.0	5.4
Liquid phase	6.6	8.3	1.1
Gas phase	71.4	77.7	93.5
Gas phase composition			
Hydrogen	3.3	1.8	2.1
Carbon monoxide	15.5	12.9	9.7
Carbon dioxide	12.8	10.1	7.9
Methane	24.1	16.0	20.7
Ethane	2.7	3.3	3.2
Ethylene	30.3	26.6	32.0
Propane	0.3	2.0	1.0
Propylene	4.5	15.1	16.1
Ethyne	1.8	1.2	1.0
1‐Butylene	0.2	1.8	0.1
1,3‐Butadiene	0.6	1.4	1.1
Benzene	1.4	0.2	0.2
Others (containing butane, allene, 2‐butene, isobutylene, propyne, etc.)	2.7	7.7	4.9

Since PE, PP, and PET account for the most plastic waste, and PET is widely recycled with mechanical processes, PE and PP were selected to test in this work. A total of 25 g PE powder was mixed with 25 g palm oil (feedstock POPE), and so was PP powder (feedstock POPP). Both POPE and POPP were pyrolyzed with the same process as palm oil above. The pyrolysis product compositions are also listed in Table 1. The results of both POPE and POPP were much better than that of using palm oil alone. Solid products were reduced and gas products were increased. As for the gas phase composition, the ethylene content remained high, at 26.6 and 32.0 wt% respectively, and the propylene content increased from 4.5 to 15.1 wt% and to 16.1 wt%, respectively. It is exciting to find that the sum of ethylene and propylene yield from POPP is similar to that from naphtha in traditional steam cracking, close to 45 wt% of raw materials. Renewable ethylene and propylene can be obtained from plastic wastes and plant oil at a reasonable cost. In addition to ethylene and propylene, other chemicals obtained by this process are all useful. Hydrogen is a valuable and clean energy resource; carbon monoxide along with water can also be converted to hydrogen; carbon dioxide can be converted to ethanol by fermentation;[qv: 12] and methane can be directly used as fuel. The polycyclic aromatic hydrocarbons and biocarbon can be used as feedstocks for chemical industry and renewable reinforcing filler for rubber industry. If PS foam or/and PET bottle are part of the source for feedstock, styrene and other aromatic hydrocarbons can also be recovered. Apparently, high temperature facilitates the decomposition of the palm oil and the plastic scraps to small molecules. Different combination of plastic wastes and plant oils for the feedstock mixture will involve different chemical reactions. Therefore, more renewable chemicals could be obtained after in‐depth study on this new process.

Based on the above new finding, conceptual “drop‐in” process was proposed for a renewable and circular polymer industry, as shown in **Figure** **2**. The process starts from cracking a mixture of palm oil and plastic wastes. The obtained pyrolysis gas can be separated into different chemicals, and some of them such as ethylene, propylene, and styrene can be polymerized into plastics again with the help of existing petrochemical technologies. This new process can achieve two things by one stroke: renewable and circular, a promising solution for both biorefinery and circular plastic industry.

**Figure 2:**
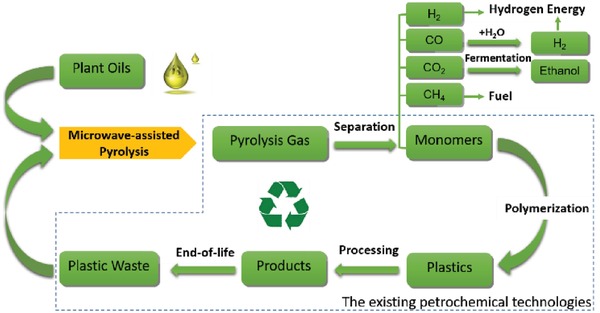
Schematic process of renewable plastics and circular chemical industry.

Chemical industry is one of major energy consumers and carbon dioxide emission sources. For example, the U.S. chemical industry accounts for 25% of manufacturing energy use and 2.6% of the country's carbon emissions.[qv: 13] Currently, more than 90% of the raw materials for chemical industry are fossil feedstocks including crude oil, shale gas, and coal.[qv: 14] Considering the dwindling fossil resources, global climate change, and increasing importance of sustainability, both chemical industry and academia have focused their interest on renewable resources.[qv: 15,16] Plant oils, especially palm oils, are extremely important renewable feedstocks for chemical industry due to their reasonable cost and large‐scale availability.[qv: 14] Chemical industry can become renewable and circular if plant oils and plastic waste can replace fossils in chemical industry. Our research results reported here make it possible.

In summary, a continuous lab‐scale approach for chemical recycling of plastics has been developed by using microwave‐assisted high temperature instead of catalysts. The mixture of plastic waste and plant oil can be cracked into ethylene, propylene, and other useful chemicals without costly and time‐intensive sorting and pretreating. This experimental technique is suitable not only for thermoplastic, but also for thermoset, rubber, and virtually all kinds of organic wastes in all phases and shapes, including fiber, composites, oil wastes, etc. The method delivered interesting leads that provide the basis for developing a new process. Based on the results of this method, a “drop‐in” method for a renewable and circular polymer industry is also proposed. If it is commercially realized, plastic waste and plant oils will be the feedstock for the polymer industry. The industry will become renewable and in line with the vision of circular economy. We hope that this report will contribute to combating plastic pollution and help plastics stay in the economy and out of the oceans.[qv: 17]

## Conflict of Interest

The authors declare no conflict of interest.

## Author Contributions

H.J. and W.L. contributed equally to this work. J.Q. contributed to conceptualization and supervision. H.J. and W.L. contributed to investigation, methodology, and writing—original draft. X.Z. contributed to data curation and writing—review and editing.

## Supporting information

Supporting Information

Supplemental Movie 1

Supplemental Movie 2
