# Supplementary material for: Chemical Recycling of Plastics by Microwave‐Assisted High‐Temperature Pyrolysis
Source: Glob Chall. 2020 Feb 14;4(4):1900074. doi: 10.1002/gch2.201900074 (PMC7117841; doi:10.1002/gch2.201900074)
Supplement: Supplementary file 1 — Supporting Information [file GCH2-4-1900074-s001.pdf]

Copyright WILEY-VCH Verlag GmbH & Co. KGaA, 69469 Weinheim, Germany, 2020.

Supporting Information

for *Global Challenges,* DOI: 10.1002/gch2.201900074

Chemical Recycling of Plastics by Microwave-Assisted High-Temperature Pyrolysis

*Haibin Jiang, Wenlu Liu, Xiaohong Zhang, and Jinliang Qiao**

Copyright WILEY-VCH Verlag GmbH & Co. KGaA, 69469 Weinheim, Germany, 2020.

Supporting Information

Chemical Recycling of Plastics by Microwave-assisted High Temperature Pyrolysis

*Haibin Jiang, Wenlu Liu, Xiaohong Zhang, Jinliang Qiao**

Table of Contents

This PDF file includes:

Materials and Methods
Figs. S1 to S5
Captions for Movies S1 to S2

Other Supplementary Materials for this manuscript include the following:

Movies S1 to S2

Experimental Procedures

Materials and Equipment

The aqueous graphene oxide (GO) suspension and the aqueous carbon nanotubes (CNTs) dispersion were purchased from Nanjing JCNANO Technology Co., Ltd. Melamine foam (MF) was supplied by BASF Applied Chemical Co., Ltd. The MF was cut into small cuboid (6 cm × 4 cm× 1 cm), washed by deionized water by sonicating for 15 min, and then dried in a vacuum oven at 50 °C. Water-soluble starch was purchased from Yu Feng Starch Co., Ltd. Alumina fiber board was purchased from Wei Ye Crystal Fiber Co., Ltd. The aluminum oxide fiber board (AF) was cut into small cuboid (5 cm × 2.5 cm× 0.5 cm), washed by deionized water by sonicating for 15 min, and then dried in a vacuum oven at 50 °C. Polyethylene powder and polypropylene powder were both supplied by SINOPEC Maoming Company. The household microwave oven was made by Galanz Microwave Oven and Electric Appliances Manufacturing Co., Ltd. The microwave reactor (MKX-R1C1B) was made by Makewave Research Institute of Microwave Physical Chemistry.

Preparation of graphene foam (GF)

The MF was put into the aqueous GO suspension of 5.0 mg/mL and squeezed five times to make sure that the MF was sufficiently wetted with the GO suspension, and then the watery GO−MF was heated in a blast oven at 100 °C for 3 h for pre-reduction. Then, the pre-reduced GO−MF was placed in a sealed quartz glass box filled with nitrogen and protected by a sealed polypropylene box. The whole box was heated in a household microwave oven at 700 W for 40 s.

Preparation of carbon nanotubes foam (CNTF)

The MF was put into the aqueous CNTs dispersion of 5.0 mg/mL and squeezed five times to make sure that the MF was sufficiently wetted with the CNTs dispersion, and then the watery CNTs−MF was heated in a blast oven at 100 °C for 3 h. Then, the CNTs−MF was placed in a sealed quartz glass box filled with nitrogen and protected by a sealed polypropylene box. The whole box was heated in a household microwave oven at 700 W for 40 s.

Preparation of carbon-coated AF (CAF)

Aqueous starch solution of 20 mg/mL was prepared. The AF was put into the prepared starch solution, and the solution would be sucked in spontaneously. The watery starch-AF was heated in a blast oven at 100 °C for 3 h. Finally, the dried starch-coated AF was carbonized at 1000℃ for 1 h in a tubular horizontal furnace, using a heating rate of 3℃·min-1 and a high-purity nitrogen flow rate of 200 ml·min-1.

Microwave-assisted pyrolysis of palm oil

0.5g palm oil was dropped on 0.2g GF or CNTF to soak. The GF or CNTF with oil was placed in an uncovered quartz glass box. Wrapped in heat insulator, the quartz glass box was sealed in a nitrogen-filled bag. The whole bag was heated in a household microwave oven at 700 W for 40 s. Thereafter, the gas in the bag was taken by a 100ml syringe for GC analysis.

Microwave-assisted pyrolysis of plastics

About 0.5g plastics scraps were placed on 0.2g GF, 0.2g CNTF, or 2g CAF, respectively. The GF, CNTF, or CAF with plastics scraps was placed in an uncovered quartz glass box. Wrapped in heat insulator, the quartz glass box was sealed in a nitrogen-filled bag. The whole bag was heated in a household microwave oven at 700W for 40s.

Continuous process for microwave-assisted pyrolysis of plant oil

Palm oil as feedstock is continuously pumped into microwave cavity through a quartz tube at the feed rate of 0.5 g/min and pyrolyzed to gas with 900W microwave and 30g CAF. After condensation, the pyrolysis gas is collected for GC analysis.

Continuous process for microwave-assisted pyrolysis of mixture of plastic waste and plant oil

25g PE powder was mixed with 25g palm oil (feedstock POPE), and POPE was continuously pumped into microwave cavity through a quartz tube at the feed rate of 0.5 g/min and pyrolyzed to gas with 900W microwave and 30g CAF. After condensation, the pyrolysis gas was collected for GC analysis. So was PP powder (feedstock POPP).

Gas products analysis

The gas products were analyzed according to ASTM D1945-14 method using a Refinery Gas Analyzer (HP Agilent 7890 A, configured with three channels, including one FID and two TCD (thermal conductivity detector)). Hydrocarbons were analyzed on the FID channel. One TCD with nitrogen carrier was employed to determine hydrogen because of the small difference in the conductivity of hydrogen and helium carrier. The other TCD with helium as carrier gas was used to detect CO, CO2, N2 and O2. For quantitative analysis, the response factor was determined by using a RGA (Refinery Gas Analysis) calibration gas standard.

Results and Discussion

Figure S1. SEM image of GF.

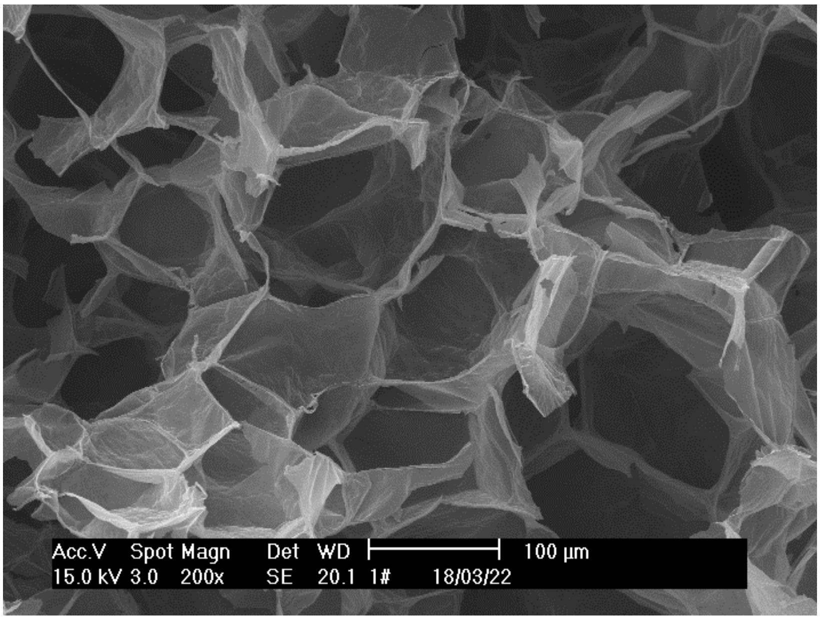

Figure S2. Photographs of plastics before (the left) and after (the right) microwave pyrolysis.

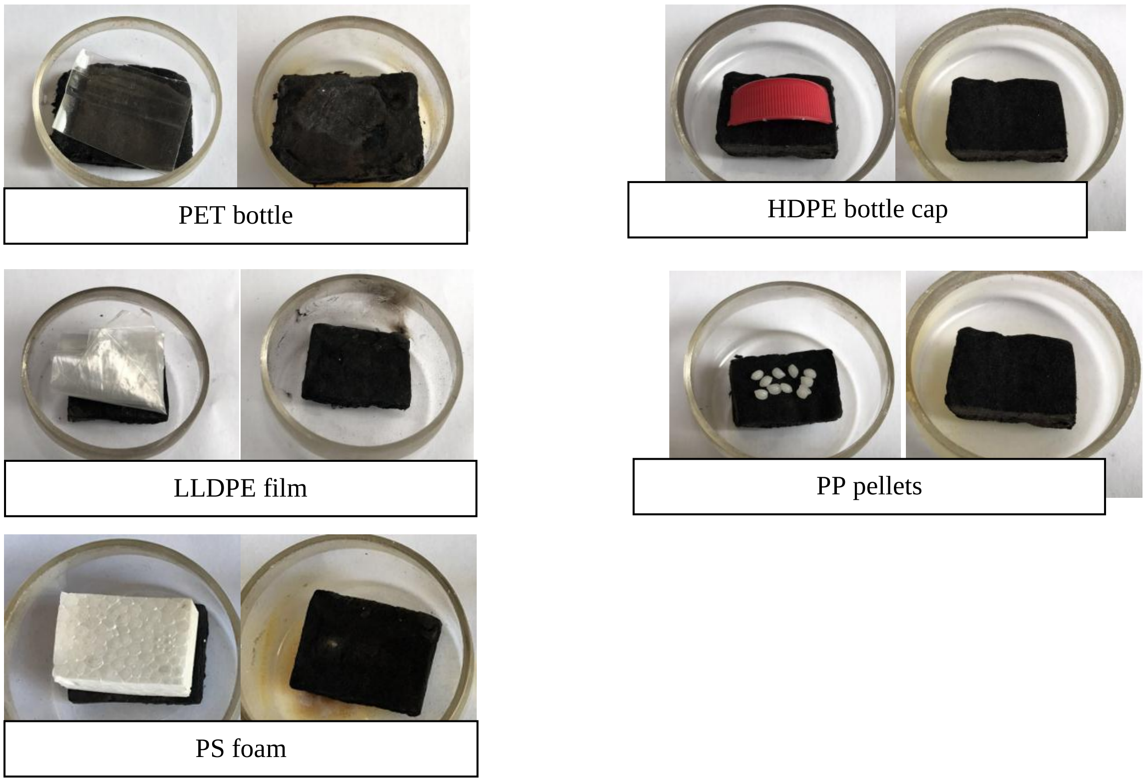

Figure S3. SEM images of CNTF.

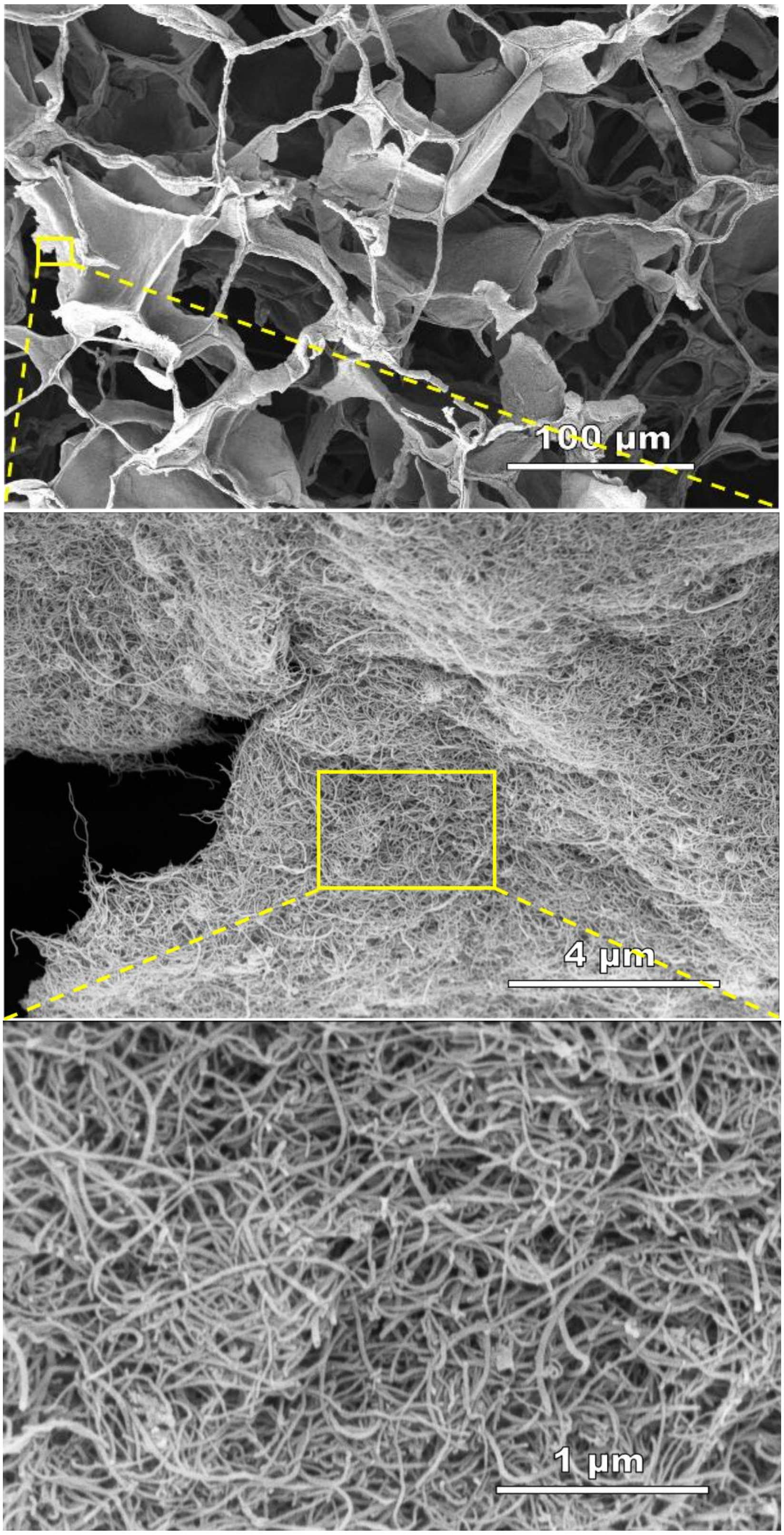

Figure S4. SEM images (the left) and SEM EDS maps (the right) of (A) AF and (B) CAF (Al map: the green; C map: the yellow).

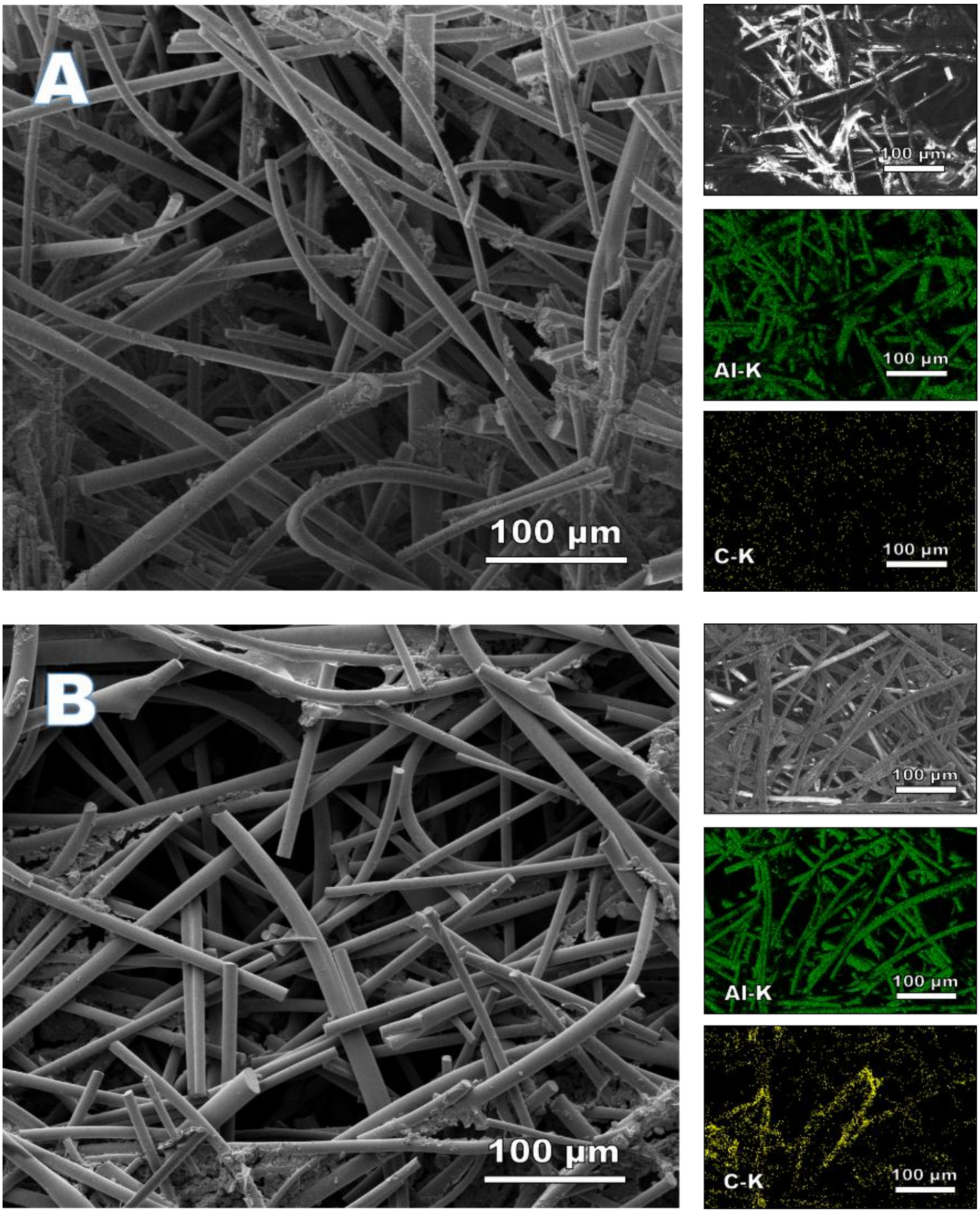

Figure S5. Temperature curve of CAF with 900W microwave irradiation..

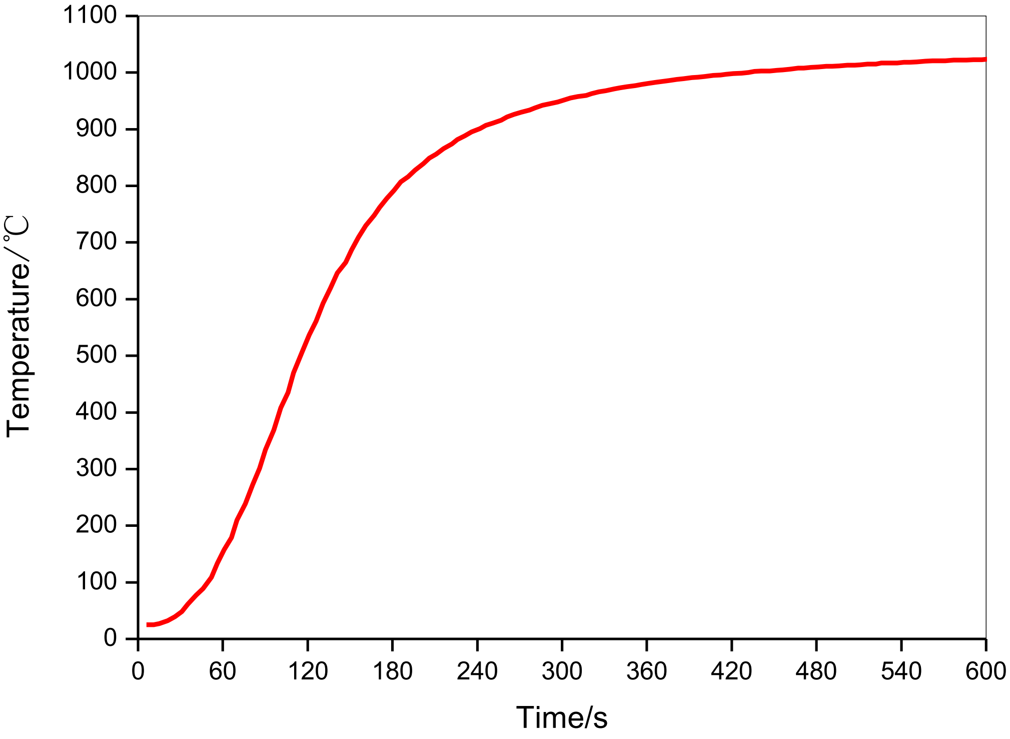

Movie S1.

Intensive arcs observed when heating GF in household microwave oven.

Movie S2.

Continuous process for microwave-assisted pyrolysis of POPE.
